# Effects of Long-Term Methotrexate, Infliximab, and Tocilizumab Administration on Bone Microarchitecture and Tendon Morphology in Healthy Wistar Rats

**DOI:** 10.7759/cureus.14696

**Published:** 2021-04-26

**Authors:** Frideriki Poutoglidou, Chryssa Pourzitaki, Maria Eleni Manthou, Efthimios Samoladas, Foteini Malliou, Athanasios Saitis, Dimitrios Kouvelas

**Affiliations:** 1 Department of Clinical Pharmacology, Aristotle University of Thessaloniki, Thessaloniki, GRC; 2 Laboratory of Histology and Embryology, Aristotle University of Thessaloniki, Thessaloniki, GRC; 3 Division of Orthopaedics, Genimatas Hospital, Aristotle University of Thessaloniki, Thessaloniki, GRC

**Keywords:** methotrexate, infliximab, tocilizumab, rheumatic diseases, bone mineral density

## Abstract

Objective

Rheumatic diseases are associated with bone loss, both systemic and periarticular, and tendon abnormalities. The aim of this study is to examine the effect of three antiarthritic drugs, methotrexate, an anti-folate metabolite; infliximab, a Tumor Necrosis Factor-alpha (TNF-α) inhibitor; and tocilizumab, an antibody against Interleukin-6 (IL-6) receptor, on bone microarchitecture and tendon morphology in the absence of an inflammatory state.

Materials and methods

Thirty-five, 8- to 9-week-old, male, Wistar rats were randomly allocated into five groups: negative control (CTRL), vehicle (VEH), methotrexate (MTX), infliximab (INFX), and tocilizumab (TCZ). After 8 weeks of antiarthritic drug intraperitoneal administration, animals were euthanized and rat tibiae and patellar tendons were histologically examined.

Results

All sections exhibited normal bone microarchitecture. Histological scores in all groups corresponded to normal bone mineral density. No no apparent differences in tenocyte morphology and architecture of collagen fibers were observed.

Conclusions

The results of this study indicate that long-term administration of methotrexate, infliximab, and tocilizumab had no effect on bone microarchitecture and tendon morphology in rats in the absence of an inflammatory condition.

## Introduction

Osteoporosis is a chronic condition characterized by a decrease in Bone Mineral Density (BMD) and a disruption of bone microarchitecture, predisposing patients to an increased risk of fragility fracture. Osteoporosis is a prevalent comorbidity in patients with rheumatic diseases, such as rheumatoid arthritis, ankylosing spondylitis, psoriatic arthritis, and juvenile idiopathic arthritis [1,2].

Rheumatic diseases are associated with both periarticular and systemic bone loss. The mechanisms underlying the localized bone loss in rheumatic diseases are not yet fully elucidated. It seems, however, to be directly related to pro-inflammatory cytokines released by the inflamed synovium, as well as the increased vascularity and immobility of the affected joints [3]. Meanwhile, the pathogenesis of generalized bone loss predominantly involves the systemic effects of inflammation, immobilization, nutritional problems, and weight loss in patients with rheumatic diseases [2,4].

Common extra-articular manifestations of rheumatic diseases include tendon abnormalities such as tenosynovitis, tendinosis, tendinitis, peritendinous inflammation, and tendon rupture [5]. Pro-inflammatory cytokines, including Tumor Necrosis Factor-alpha (TNF-α) and Interleukin-6 (IL-6), are implicated in the development of tendon damage in the inflammatory spectrum [6].

Methotrexate, one of the most commonly used disease-modifying antirheumatic drugs (DMARDs), is an anti-folate metabolite, which acts by inhibiting the enzyme dihydrofolate reductase, thus affecting deoxyribonucleic acid (DNA) synthesis and cell proliferation. Infliximab is one of the most widely used TNF-α inhibitors that has been approved for the treatment of a variety of inflammatory diseases, such as rheumatoid arthritis, ankylosing spondylitis, and psoriatic arthritis. Tocilizumab is a monoclonal antibody against the IL-6 receptor (IL-6R) that is mainly used for the treatment of juvenile idiopathic arthritis and rheumatoid arthritis.

Previous studies have demonstrated a beneficial effect of TNF-α blockade on bone metabolism in patients with rheumatic diseases [7-9]. Interleukin-6 inhibition has been studied to a lesser extent. A number of studies have suggested that tocilizumab might prevent bone loss associated with rheumatic diseases [10]. Methotrexate has been linked with a loss of bone density in oncology patients [11]. Nevertheless, low-dose regimens for inflammatory diseases do not seem to negatively affect bone turnover [12,13].

It has not yet been established whether biologic agents prevent bone loss in rheumatic diseases through a direct mechanism on bone cell metabolism or indirectly by suppressing inflammation. Only a few studies have examined the differences in bone markers in relation to biologic response [14,15]. Moreover, the bone loss induced by the disease itself may conceal the real effects of the drugs on bone metabolism. On the other hand, the effect of TNF-α and IL-6 depletion on tendon tissue remains to be investigated. The aim of this study is to evaluate the effect of long-term administration of three commonly used antiarthritic drugs -- methotrexate, infliximab, and tocilizumab -- on bone microarchitecture and tendon morphology in healthy rats without an inflammatory condition.

## Materials and methods

Animals

The study was approved by the Directorate of Veterinary Services of the Region of Central Macedonia according to national legislation (Presidential Decree 56/2013, in conformance with the European Directive [2010/63/EU] [reference number: 668476(3484] 21643[87], 06/10/2019). Twenty-eight 7- to 8-week-old male Wistar rats (250-300g) were provided by the Animal Facility of the Department of Pharmacology of the National and Kapodistrian University of Athens. All rats were housed in the animal house of our laboratory in a specific pathogen-free environment to constant temperature 21-22°C, relative humidity 50-60%, and a light/dark cycle of 12/12 hours (lights on at 07:00 am). The animals were placed in Plexiglas chambers in groups of three to four and ad libitum access to standard rodent pellet diet and water was provided at all times. Before the study, the rats were allowed a one-week acclimatization period to recover from shipping-related stress.

Drug administration

Following the habituation period, the rats were randomly allocated into five groups, each comprising seven rats, and received all drugs intraperitoneally for 8 weeks: 1) negative control group (CTRL), 2) 0.5 mL vehicle (0.9% saline) injection, once a week (VEH), 3) methotrexate, 0.35 mg/kg, once a week (MTX) [16], 4) infliximab, 5 mg/kg, once a week (INFX) [17], and 5) tocilizumab, 8 mg/kg, once every two weeks (TCZ) [18]. The drugs were freshly prepared and the doses were selected based on the literature and preliminary studies.

Bone histology

After 8 weeks of antiarthritic drug administration, rats were anesthetized with 5% isoflurane and euthanized by decapitation. Rat bones (tibia diaphyses) were harvested, fixed in 4% formaldehyde, decalcified by 5% nitric acid for four days, enclosed in paraffin blocks, and cut longitudinally, using a Leica microtome (Leica Biosystems, Wetzlar, Germany), into 5-6 μm thick sections. The sections were stained with hematoxylin and eosin (H&E) stain and studied under a light Zeiss Primo Star microscope (Carl Zeiss, Oberkochen, Germany). A Canon A620 camera (Canon, Tokyo, Japan) was used to take photographs of each specimen.

We used the grading system of bone affection adopted by Khalifa et al. [19], which was established based on the scoring protocol of Pritzker et al. [20]. For each sample, the following parameters we assessed: thickness of the cortical bone, vascularity, size of the cells, number of cells, matrix homogenicity, arrangement of osteons, and periosteal irregularity. A mean value was documented for each of the seven parameters, in every group of animals. The mean values in each group were added and the total score ranged from 0 to 21. Scores ranging from 0-4 were the best assessment and were considered normal. Scores from 5-10 corresponded to mild grade osteoporosis, scores from 11-16 to moderate grade, and scores from 17-21 to severe osteoporosis.

Tendon histology

Rat patellar tendons were harvested, fixed in 4% formaldehyde for a day before being treated with ethanol and xylene. They were enclosed in paraffin blocks and cut longitudinally using a Leica microtome into 5-6 μm thick sections. The sections were dyed with H&E stain and were studied under a light Nikon Eclipse E600 microscope (Nikon, Tokyo, Japan). A Nikon D5-Fi1 camera (Nikon, Tokyo, Japan) was used to take photographs of each specimen.

## Results

**Figure 1:**
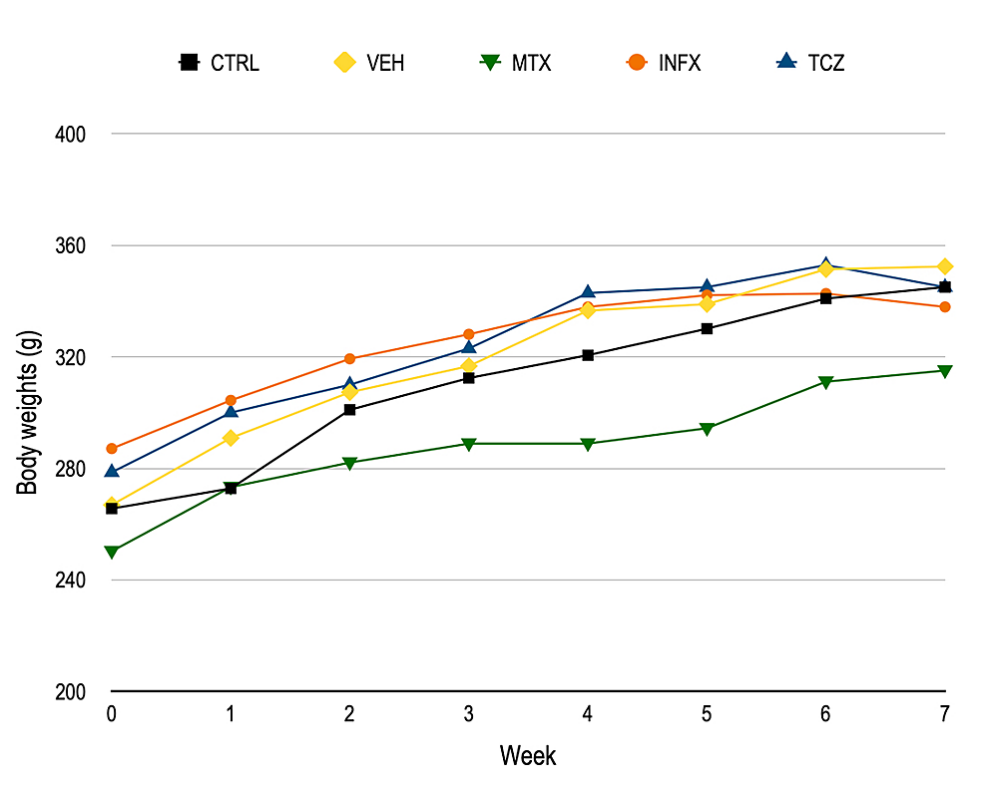
Changes in body weight throughout the experiments. Each value represents the mean. No significant differences were observed among the groups. CTRL: negative control group; VEH: 0.5 mL vehicle (0.9% saline) injection, once a week; MTX: methotrexate, 0.35 mg/kg, once a week; INFX: infliximab, 5 mg/kg, once a week; TCZ: tocilizumab, 8 mg/kg, once every two weeks.

Body weights increased in a comparable manner between the groups. No significant differences were observed between non-treated or vehicle-treated animals and animals receiving treatment (Figure 1).

**Figure 2:**
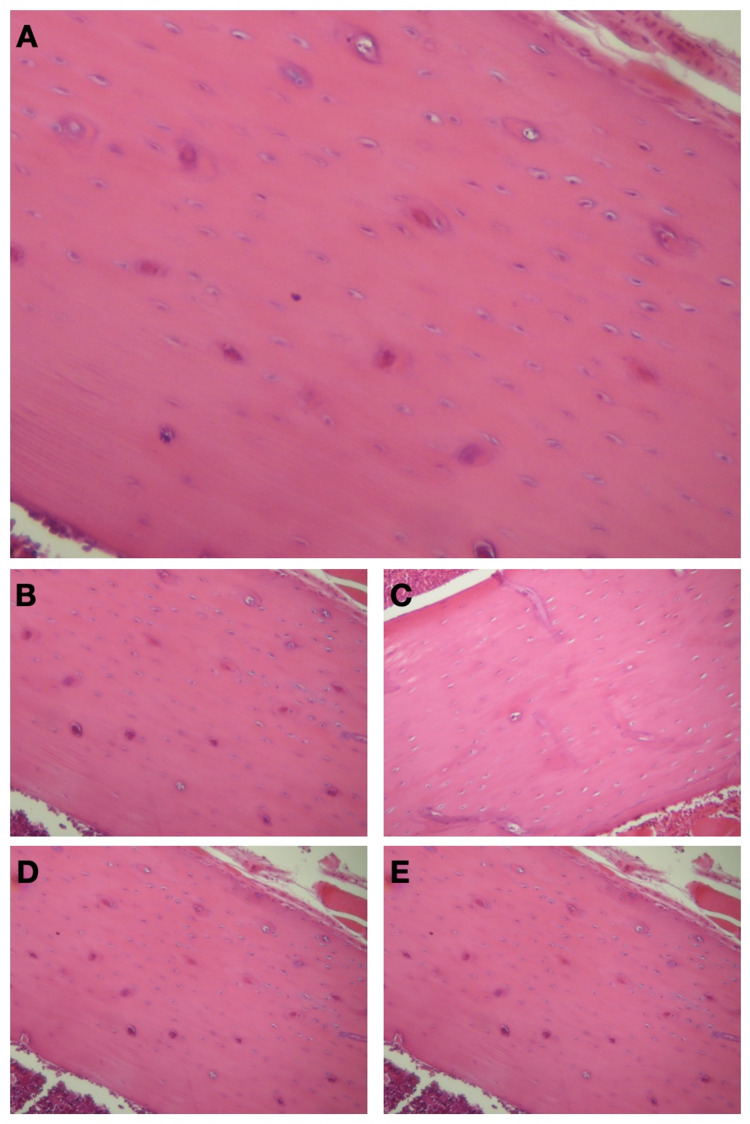
Representative H&E sections of the rat tibiae in the five groups. A. CTRL (X400), B. VEH (X400), C. MTX (X400), D. INFX (X400), E. TCZ (X400). All sections exhibited normal bone microarchitecture. CTRL: negative control group; VEH: 0.5 mL vehicle (0.9% saline) injection, once a week; MTX: methotrexate, 0.35 mg/kg, once a week; INFX: infliximab, 5 mg/kg, once a week; TCZ: tocilizumab, 8 mg/kg, once every two weeks.

Microscopical study of the sections revealed no differences between the groups. All sections exhibited normal microarchitecture. CTRL- and VEH-treated animals had a similar thickness of cortical bone, size, and number of cells compared to all treatment groups (MTX, INFX, TCZ). No differences were noted in terms of matrix homogenicity, arrangement of osteons, or periosteal irregularity. Histological scores in all groups corresponded to normal bone mineral density (0-4). Representative sections of H&E staining of rat tibiae in the five groups are shown in Figure 2.

**Figure 3:**
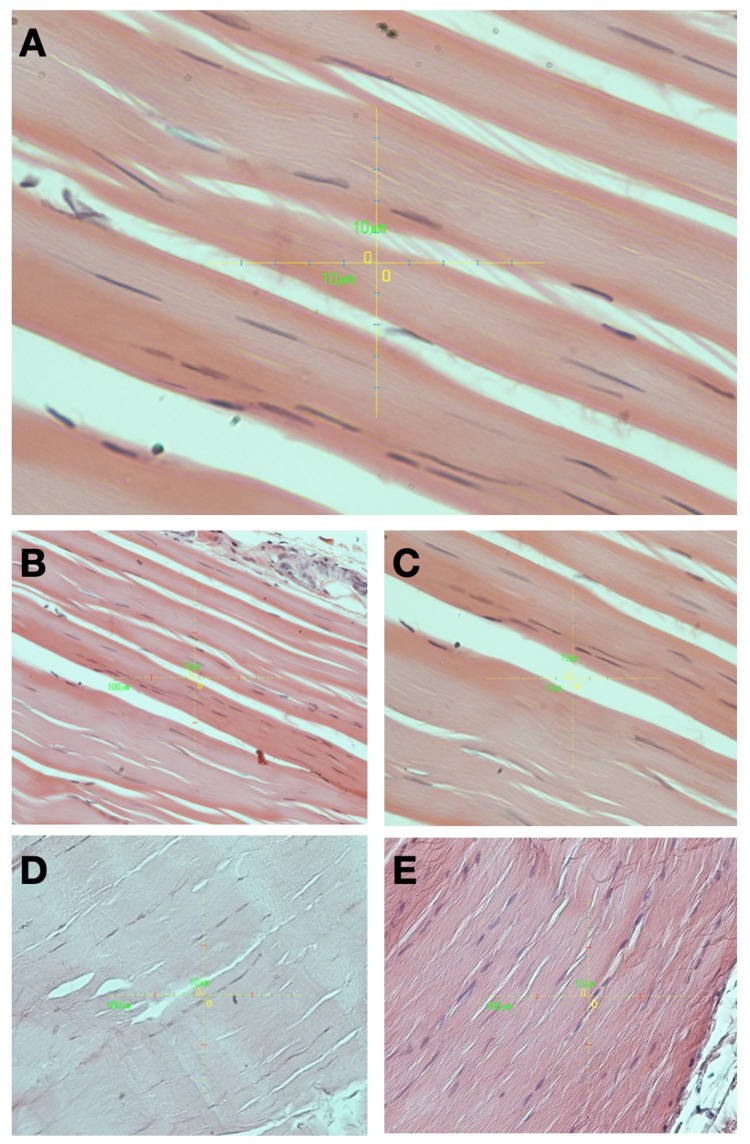
Representative H&E sections of rat patellar tendons in the five groups. A. CTRL (X40). B. VEH (X40). C. MTX (X40). D. INFX (X40). E. TCZ (X40). Collagen fibers appear with no apparent disruptions or disturbances in architecture. They are aligned parallel to each other in a wavy manner. Arranged between them are long, thin tenocytes. CTRL: negative control group; VEH: 0.5 mL vehicle (0.9% saline) injection, once a week; MTX: methotrexate, 0.35 mg/kg, once a week; INFX: infliximab, 5 mg/kg, once a week; TCZ: tocilizumab, 8 mg/kg, once every two weeks.

Microscopical study of longitudinally sectioned tendons from all groups of animals revealed no apparent differences in tenocyte morphology and architecture of collagen fibers. Collagen fiber bundles appeared with no apparent disruptions or disturbances in architecture. Fibers were aligned parallel to each other, throughout the specimen, in a wavy manner. Interposed between them, long, thin tenocytes were observed, evenly distributed throughout the specimen, and arranged in longitudinal rows. Representative sections of H&E staining of rat patellar tendons in the five groups are presented in Figure 3.

## Discussion

Bone homeostasis is maintained by a balance between bone resorption by osteoclasts and bone formation by osteoblasts. Chronic inflammation leads to the secretion of a plethora of pro-inflammatory cytokines, such as TNF-α, Interleukin-1 (IL-1), and IL-6. Receptor activator of nuclear factor-kappa Β ligand (RANKL), a transmembrane protein belonging to the TNF-α superfamily, is essential for osteoclastogenesis and osteoclast activation. RANKL expression is stimulated by pro-inflammatory cytokines produced by synovial fibroblasts in inflamed joints [21]. Although the increase in osteoclastic activity appears to be the main mechanism of inflammation-related bone loss, it has been shown that TNF-α may inhibit osteoblast-mediated bone formation via upregulation of the Wnt signaling inhibitors, Dickkopf-related protein-1 (DKK-1), and sclerostin [22].

Experimental studies on animal models of arthritis have indicated a positive effect of TNF-α inhibition on bone metabolism [23]. It has also been reported that IL-6 signaling blockade by an anti-IL6R antibody can prevent the bone loss induced by collagen-induced arthritis (CIA) animal model [24]. In our study, we demonstrate that both TNF-α and IL-6 blockade has no effect on bone microarchitecture in healthy rats. This supports the hypothesis that TNF-α and IL-6 inhibition has a facilitatory effect on bone metabolism only in the presence of an inflammatory state. It is likely that the improvement in bone markers reported in previous studies might not be a result of neutralization of TNF-α or IL-6 per se, rather are simply related to the improvement of disease activity secondary to reduction of the inflammation status.

It has been shown that high dose methotrexate administration in rats at a dose of 0.75 mg/kg in five-day courses, corresponding to a chemotherapy regimen in human patients, had a negative effect on bone metabolism [25]. A decline in mineral density was also observed when methotrexate was administered at a dose of 0.5 mg/kg twice-weekly [26]. On the other hand, lower doses ranging from 0.2 mg/kg to 0.4 mg/kg did not seem to affect bone metabolism significantly [25,27]. We found that methotrexate administration, at a dose of 0.35 mg/kg weekly, had no effect on bone microarchitecture. This confirms the view that methotrexate-induced bone loss is largely dependent on treatment dose and regimen.

It has been shown that TNF-α can strongly activate tenocytes and stimulate cytokine production that further inhibits extracellular matrix synthesis [28]. IL-6 is highly expressed in ruptured human tendons mainly around proliferative vessels [29], while tendon fibroblasts secrete IL-6 at an increased level in response to cyclical stretching [30]. The results of our study show that TNF-α and IL-6 inhibition has no effect on tendon morphology in rats in the absence of an inflammatory condition.

## Conclusions

In conclusion, the results of this study indicate that long-term administration of methotrexate, infliximab, and tocilizumab had no effect on bone microarchitecture and tendon morphology in healthy rats. This is a negative study; it does, however, provide further insights into the mechanism of action and potential side effects of three commonly used antiarthritic drugs. The present study investigates the effects of treatment on the histological appearance of bone and tendon tissue. Future studies on the current topic are required to investigate the effects of these agents at a molecular level.
